# Integrative multiomics analysis identifies a metastasis-related gene signature and the potential oncogenic role of EZR in breast cancer

**DOI:** 10.32604/or.2022.026616

**Published:** 2022-12-06

**Authors:** GUODONG XIAO, FENG CHENG, JING YUAN, WEIPING LU, PEILI WANG, HUIJIE FAN

**Affiliations:** 1 Department of Oncology, The First Affiliated Hospital of Zhengzhou University, Zhengzhou, 450052, China; 2 Breast Cancer Center, Affiliated Cancer Hospital of Zhengzhou University, Henan Cancer Hospital, Zhengzhou, 450008, China

**Keywords:** Metastasis, Gene signature, EZR, Breast cancer

## Abstract

Distant metastasis is a major cause of increased mortality in breast cancer patients, but the mechanisms underlying breast cancer metastasis remain poorly understood. In this study, we aimed to identify a metastasis-related gene (MRG) signature for predicting progression in breast cancer. By screening using three regression analysis methods, a 9-gene signature (NOTCH1, PTP4A3, MMP13, MACC1, EZR, NEDD9, PIK3CA, F2RL1 and CCR7) was constructed based on an MRG set in the BRCA cohort from TCGA. This signature exhibited strong robustness, and its generalizability was verified in the Metabric and GEO cohorts. Of the nine MRGs, EZR is an oncogenic gene with a well-documented role in cell adhesion and cell migration, but it has rarely been investigated in breast cancer. Based on a search of different databases, EZR was found to be significantly more highly expressed in both breast cancer cells and breast cancer tissue. EZR knockdown significantly inhibited cell proliferation, invasion, chemoresistance and EMT in breast cancer. Mechanistically, RhoA activation assays confirmed that EZR knockdown inhibited the activity of RhoA, Rac1 and Cdc42. In summary, we identified a nine-MRG signature that can be used as an efficient prognostic indicator for breast cancer patients, and owing to its involvement in regulating breast cancer metastasis, EZR might serve as a therapeutic target.

## Introduction

Breast cancer (BC) is the most frequent malignancy and the second leading cause of cancer-related mortality in females worldwide. According to estimates, approximately 2,088,849 new BC cases and 626,679 deaths occurred in 2018 worldwide [1]. Although the incidence rate is higher in developed countries, overall mortality is greater in developing countries. BC survival rates range from over 80% in many developed countries to below 40% in developing countries [2]. Overall, the mortality rates of BC have decreased with improvements in therapeutic strategies. Unfortunately, approximately 15% of patients are initially diagnosed with incurable disease at an advanced stage, and nearly 30% of women diagnosed with early-stage BC will eventually develop metastasis. Indeed, metastatic invasion to organs such as the bone marrow and lung is the major cause of death for BC patients. Therefore, advanced BC with distant metastasis is still a therapeutic challenge, and an effective prognostic prediction model is urgently needed for BC patients.

Metastasis is considered a lethal step in the progression of BC, leading to the breakdown of physiological homeostasis. Despite the high heterogeneity and epigenetic aberrations occurring in BC, several biological factors for prognosis evaluation in metastatic BC have been identified by multiomics analysis. These include matrix metalloproteinase 2 (MMP2) [3], CD44 [4] and MDM2 [5], and metastasis-related genes (MRGs) may act as significant prognostic biomarkers for patients with BC. Furthermore, several gene signatures have been constructed to guide prognosis prediction in BC. Compared to individual clinical variables, a signature with an optimal combination of candidate biomarkers significantly improves the accuracy and stability of prediction. However, signatures are largely not used clinically. Thus, it is meaningful to identify a novel and robust gene signature with promising clinical utility in BC.

Over the past few years, the emergence of high-throughput technologies has revolutionized the analysis of cancer research involving the genome and transcriptome. Through analysis of high-throughput sequencing data from public databases, some MRG signatures associated with survival prognosis have been identified in many tumor types, such as hepatocellular carcinoma [6], osteosarcoma [7], and pancreatic ductal adenocarcinoma [8]. For example, by integrating data in multiple public databases, Dou et al. identified a 7-metastasis-related lncRNA signature through analysis of lncRNA expression profiling in clear cell renal cell carcinoma patients [9]. Hu et al. [10] discovered a six-gene set associated with distant metastasis in gastric cancer patients, and Hu et al. developed a 4-MRG signature for BC patients through Gene Expression Omnibus (GEO) database analysis [11]. Conversely, there are few studies on mRNA combination biomarkers for BC metastasis using multiplatform data integration.

Motivated by these previous efforts and the need for a robust MRG signature, we identified 9 MRG markers through integrative analysis of multiomics data, including The Cancer Genome Atlas (TCGA) [12], Gene Expression Omnibus (GEO) [13] and Molecular Taxonomy of Breast Cancer International Consortium (METABRIC) [14]. Moreover, the signature was verified and analyzed by combining it with clinicopathological features. Of the nine MRGs, EZR was revealed as a potent cancer-promoting gene highly expressed in BC tissue samples. The effects of EZR on BC cell proliferation, migration, and epithelial-mesenchymal transition (EMT) were evaluated, and its involvement in the mechanism of BC metastasis deserves further study.

## Materials and Methods

### Data collection and processing

The transcriptome and clinical data of 1109 BC cases and 133 normal breast cases were extracted from TCGA (https://tcga-data.nci.nih.gov/tcga/). All primary RNA expression profile data were first normalized with the transcripts per million (TPM) method and subjected to log2(TPM+1) transformation. Gene expression and clinical data in the METABRIC BC dataset were retrieved via cBioPortal (http://www.cbioportal.org/) [15,16]. Two GEO datasets (GSE21653 and GSE20685) were obtained from the GEO website (http://www.ncbi.nlm.nih.gov/geo/). Clinical information was reviewed, and samples with incomplete information or survival duration less than 30 days were removed. A protein expression matrix for EZR was generated using CPTAC-BRCA data (https://cptac-data-portal.georgetown.edu/cptac/s/S015) [17]. Immunohistochemistry (IHC) images of EZR in normal and BC tissues were obtained from the online Human Protein Atlas database (HPAD) (https://www.proteinatlas.org/) [18]. EZR expression in 63 BC cells and 5 normal breast cells was downloaded from the Cancer Cell Line Encyclopedia (CCLE) website (http://www.broadinstitute.org/ccle/home) [19].

### Construction and validation of a prognostic MRG signature

An MRG list containing 166 genes was obtained from CancerSEA (http://biocc.hrbmu.edu.cn/CancerSEA/goDownload) [20]. A total of 164 genes were ultimately identified by intersecting TCGA and the MRG list to further construct the prognostic model. Then, univariate Cox regression was performed to screen out MRGs associated with prognosis, followed by confirming the final prognostic signature using least absolute shrinkage and selection operator (LASSO) regression and multivariate Cox regression analyses. LASSO-Cox regression was performed with the R package “glmnet”. Genes with *P* values less than 0.05 in multivariate Cox regression analysis were identified as candidate genes for prognosis. Next, the risk score for each patient was calculated according to the following formula:

$${\left(Riskscore\right)}^{}=\sum_{i=1}^{N}\left(\;Expi*Coef\;\right)$$

where N, Expi and Coef represent the gene number, gene expression level and coefficient value, respectively. Based on the cutoff point (median risk score), patients were stratified into a low-risk group or a high-risk group. To validate the specificity and sensitivity of the prognostic model, the log-rank test was performed to compare differences in overall survival (OS) between the high- and low-risk groups in multiple datasets. Kaplan‒Meier curves were drawn by using R packages (“survival” and “survminer”). The R package “pROC” was used to generate receiver operating characteristic (ROC) curves and obtain area under the curve (AUC) values. The risk curves and scatter diagrams drawn by R software (“ggplot2”) were utilized to show the risk score and the survival outcome of each BC patient in TCGA datasets.

### Correlation of clinical features and risk scores

To better understand the effect of the MRG signature on tumorigenesis and development, subgroup analysis was used to investigate the relationship between the risk score and clinical features. First, differences in the distribution of the risk scores were compared under different clinical stratifications; the results were analyzed using the Wilcoxon rank test and visualized as boxplots and heatmaps in R (“ggplot2” and “pheatmap”). Then, Kaplan‒Meier analysis was performed using the “survival” package to analyze OS differences in various clinical subgroups between the high-risk and low-risk groups. Finally, univariate and multivariate Cox regression analyses were performed to identify whether the risk score have prognostic value independent of other clinical features. The above results are displayed as a forest map. Based on the results from multivariate analyses, a nomogram for OS prediction was established by using the “rms” R package, and a calibration curve was used to estimate the prediction accuracy of the model.

### Functional analysis and protein-protein interaction (PPI) network construction

To investigate potential molecular mechanisms involved in the risk model, gene set enrichment analysis (GSEA) was performed via downloaded GSEA software (www.broadinstitute.org/gsea) [21]. GESA was implemented in the Java program language to predict biological functions associated with the risk model. The significance threshold was set at FDR < 0.25, NOM *p* value < 0.05, and |NES| > 1 after performing 1000 permutations. To explore key proteins directly related to EZR expression, a protein‒protein interaction (PPI) network was constructed by using Search Tool for the Retrieval of Interacting Genes (STRING) (https://cn.string-db.org/).

### Exploration of tumor-infiltrating immune cells (TIICs) and immune checkpoint inhibitors (ICIs) in the risk model

The CIBERSORT algorithm was utilized to calculate the estimated abundance of 22 TIICs in TCGA-BRCA datasets. The Wilcoxon nonparametric test was applied to screen for TIICs with significant differences between the high- and low-risk groups. The violin plot drawn by the R package “ggplot2” was utilized to demonstrate the above significant differences. The correlation between TIICs and the risk score was evaluated by Spearman’s correlation test (*p* < 0.05) and displayed with radar plots using the R package “fmsb”. Spearman’s rank correlation coefficient was calculated to evaluate linear correlation between the risk score and immune checkpoint-related genes. Correlation heatmaps were generated with the R package “pheatmap”.

### Assessment of the tumor microenvironment (TME) and stemness indexes of the risk score

The ESTIMATE algorithm was applied to quantify three TME scores (stromal scores, immune scores and estimate scores) using the “estimate” package in R. Two stemness indexes, the gene expression-based stemness index (mRNAsi) and DNA methylation-based stemness index (mDNAsi), were computed using a one-class logistic regression machine learning (OCLR) algorithm [22]. Relationships between the three TME scores, two stemness indexes and risk score are illustrated using boxplots and heatmaps.

### Cell culture and transfection

BC cell lines (MCF-7, MCF-7/ADR, BT-549, BT-549/ADR, MDA-MB-468 and SKBR-3) were purchased from Procell Company (Wuhan, China). MCF-7 cells were cultured in DMEM (Gibco, USA) and BT-549 cells were RPMI 1640 (HyClone, USA). MCF-7/ADR cells were grown in DMEM containing 10% FBS and1 µg/mL ADR. BT-549/ADR cells were grown in RPMI 1640 containing 10% FBS and1 µg/mL ADR. MDA-MB-468 cells were cultured in DMEM/F12 medium with 10% FBS. SKBR-3cells were plated in DMEM supplemented with 10% FBS. All media were supplemented with 10% fetal bovine serum (BI, Israel) and 1% penicillin/streptomycin (HyClone, USA). The cells were grown in a 37°C humidified incubator with 5% CO_2_. EZR siRNAs (si-EZR), scrambled negative control (NC) siRNAs (si-NC), empty pcDNA3.0 vector and EZR-pcDNA3.0 vector were synthesized by Gene Pharma (Shanghai, China), as follows: EZR 5′-UUCUCAUAAAUAUUCAGUCCAAGGG-3′, 5′-UUCUGCGTACCUAUCACGUTT-5′. Briefly, cells were plated in six-well plates at a density of 5 × 10^5^ cells/well overnight and then transfected with 50 nm si-EZR or si-NC by using Lipofectamine 3000 reagent (Invitrogen, USA).

### Quantitative real-time PCR (qRT‒PCR) analysis

Total RNA was extracted from cells by using a TRIzol RNA extraction kit (Invitrogen, USA). Total RNA was reverse transcribed into cDNA with reverse transcriptase (AMV-XL reverse transcriptase, Takara). Quantitative PCR was performed using SYBR Premix Ex Taq ii (Takara #RR820A, Japan) and a CFX Connect Real-Time System (Bio-Rad, USA). GAPDH was used as the reference gene for qPCR. The qPCR primer sequences were as follows: forward 5’-CACGCTTGTGTCTTTAGTGCTCC-3′ and reverse 5′-ACTCAGACTTTACAGGCATTTTCC-3′; GAPDH forward 5′-TGAAGGTCGGAGTCAACGGATTTGG-3′ and reverse 5′-GGAGGCCATGTGGGCCATGAG-3′. The relative expression of EZR mRNA was determined by the comparative threshold cycle (2^−∆∆Ct^) method.

### Colony formation assay

Cell proliferation ability was assessed by using the colony formation assay. In brief, cells at a density of 500 cells/well were seeded in 6-well plates after transfection and cultured in DMEM or RPMI 1640 (10% FBS) for 2 weeks. Subsequently, all colonies were fixed in 4% paraformaldehyde (Beyotime, Shanghai, China) for 10 min and stained with 0.1% crystal violet for 5 min. Colonies greater than 100 μm in diameter were scored as positive using an inverted microscope. Each sample was assessed in triplicate.

### Western blot analysis

Western blot analysis was performed as previously described. The primary antibodies and dilutions used were as follows: anti-EZR (Cat #: 3145, 1:1000, Cell Signaling) and anti-β-actin (Cat #: sc-47778, 1:1000, Santa Cruz Biotechnology). An EMT antibody sampler kit was purchased from Cell Signaling Technology (Danvers, MA, USA). HRP-conjugated secondary antibodies (anti-mouse and goat anti-rabbit) were obtained from Beyotime Biotechnology (Shanghai, China). Protein blots were visualized using enhanced chemiluminescence (ECL) reagent and analyzed with ImageJ software.

### Transwell assay

First, cells were grown to 80–90% confluence after overnight starvation and then resuspended to a density of 5 × 10^4^ cells/ml in serum-free medium. Afterward, the diluted cells were seeded into the upper chamber of a Transwell device, and 700 µl of complete medium containing 10% FBS was added to the lower chamber. After 24 h of incubation at 37°C, the bottom membrane of the chamber was washed three times in 1 × PBS buffer, and the cells were stained with 0.1% crystal violet solution. Images of invaded cells were captured with a microscope at 400× and photographed, counted and statistically analyzed.

### Rho GTPase activation assays

Activation of small GTPases, including RhoA, Cdc42, and Rac1, was assessed using an EZ-Detect Rho GTPase assay Kit (Pierce, Rockford, IL) according to the supplier’s protocols [23]. Briefly, primary MCF-7 and BT-549 cells were serum-starved overnight to reach 70–80% confluence and treated with FBS or EGF (50 ng/ml) for 5 min. GTPase activation was assessed according to the manufacturer’s instructions.

### Pull-down assays

RhoA-GTP and Rac1-GTP activities were assessed in GST-RBD and GST-PBD pull-down assays, respectively. The detailed experimental protocol was described in a previous study [24]. Briefly, cells were grown in regular media to attain 70% confluency and stimulated with EGF (50 ng/ml) for 5 min. For si-NC or si-EZR treatment, cells were preincubated overnight. Anti-RhoA (Cat: #2117, 1:200) was obtained from Cell Signaling Technology, anti-Rac1 (Cat: 05-389, 1:100) was purchased from Millipore, and the anti-GST antibody (Cat: sc-53909, 1:1000) was obtained from Santa Cruz Biotechnology, Inc. (Dallas, TX, USA).

### Statistical analysis

All statistical analyses were performed using R 4.0.3 software, and GraphPad prism5.0 software was utilized to display the data. For all processes, *p* < 0.05 was recognized as statistically significant. Comparisons were performed using the Wilcoxon-Mann-Whitney test (for two groups), the Limma test, and the Student *t*-test for paired samples showing normal distribution. For multiple groups, the Kruskal-Wallis test followed by Dunn’s multiple comparison test was used. In the figures, *p* values are provided as follows: *p < 0.05; **p < 0.01; ***p < 0.005; ****p < 0.0001.

## Results

### Construction and identification of the 9 metastasis-related gene signature

**Figure 1:**
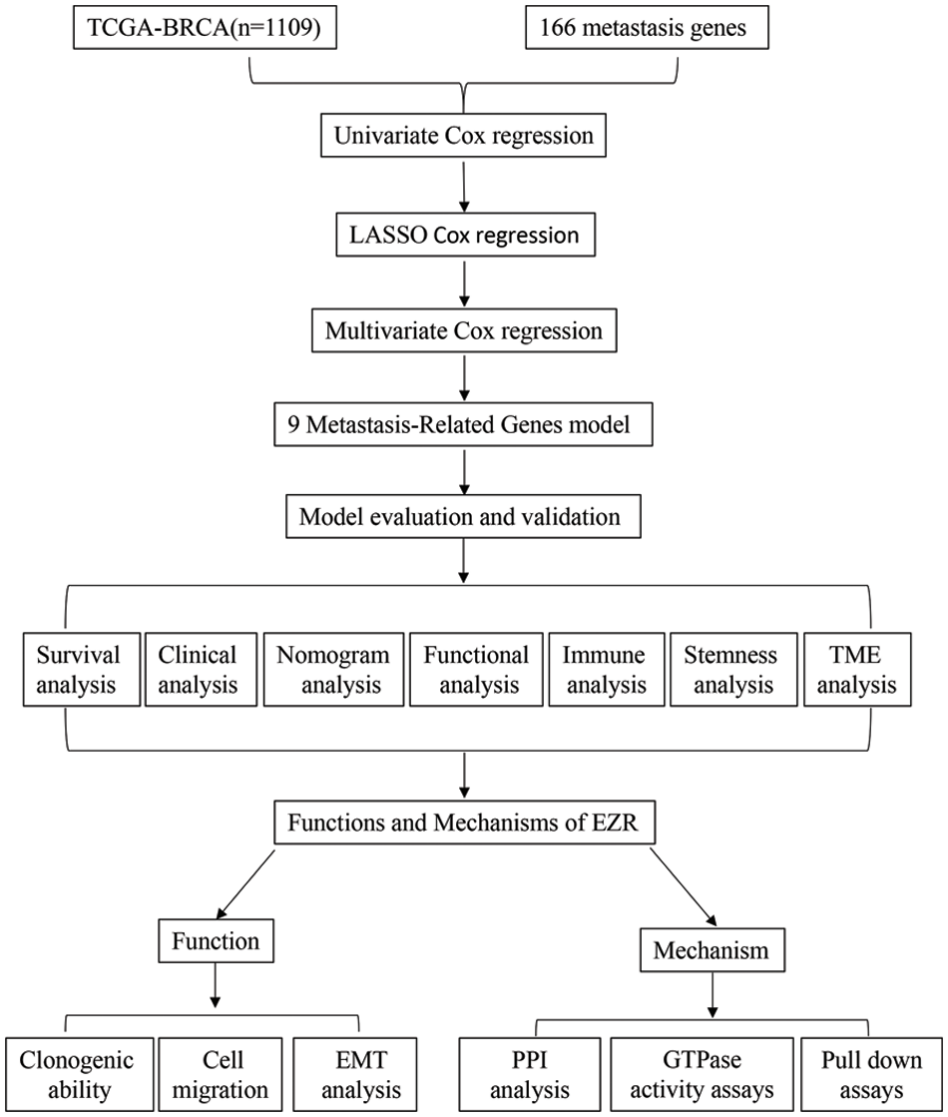
Study flow chart for the identification of a 9 MRG signature in BC.

**Figure 2:**
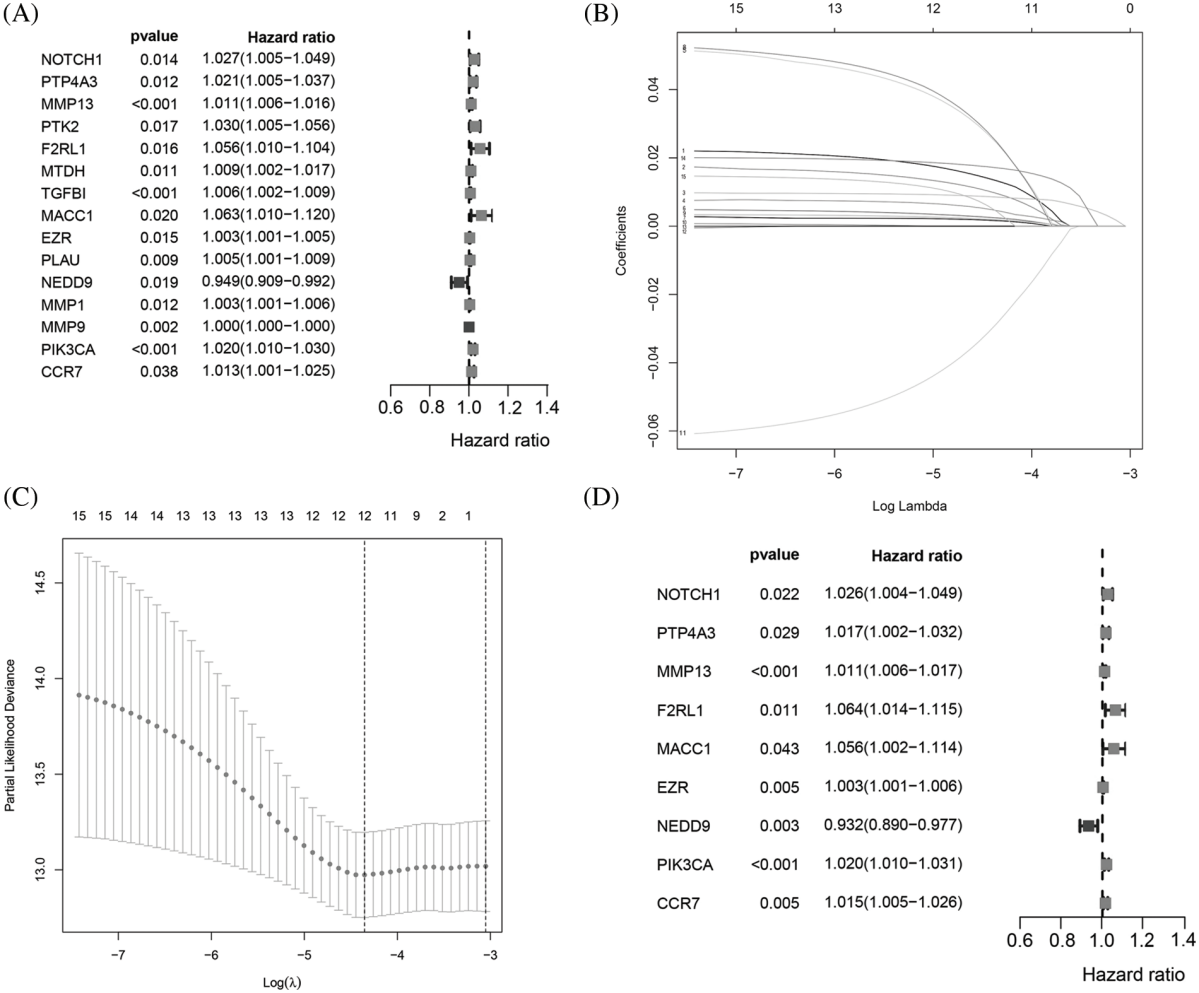
Identification of a 9MRG signature in TCGA-BRCA cohort. (A) Forest plot showing the 12 MRGs screened by univariate regression analysis. (B–C) LASSO algorithms were further performed to screen for MRGs associated with breast cancer prognosis. (D) Multivariate Cox regression analysis was performed to obtain optimal MRGs affecting prognosis.

**Supplemental Figure 1:**
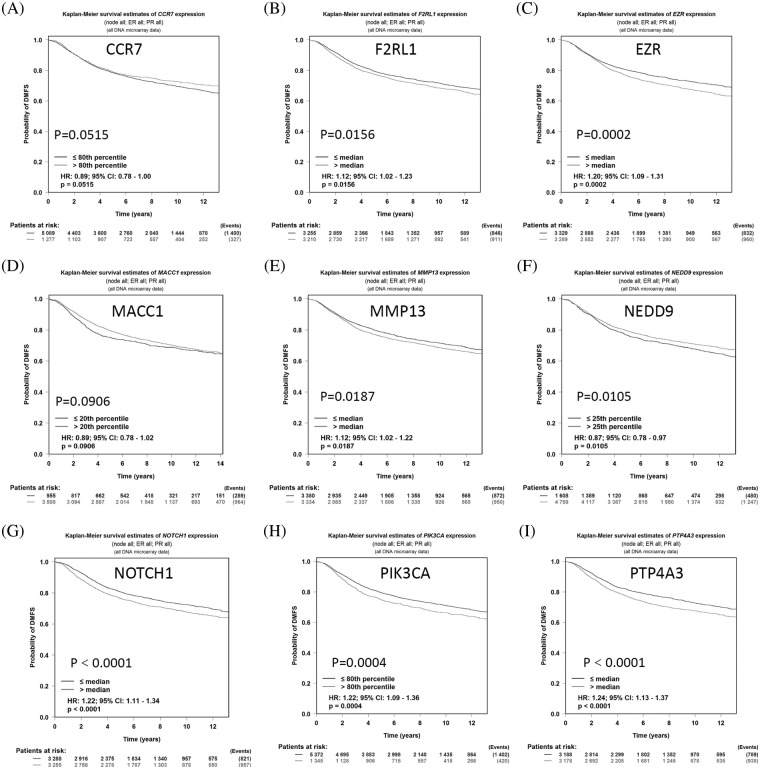
Evaluation of the 9-Metastasis-associated key genes in relationship to the OS of BC patients. Survival analysis showed the OS rates in patients with different CCR7 group (A), F2RL1 group (B), EZR group (C), MACC1 group (D), MMP13 group (E), NEDD9 group (F), NOTCH1 group (G), PIK3CA group (H), PTP4A3 group (I) according to the onlineexpression datasets (bc-GenExMiner v4.8).

The flowchart of the study is presented in Fig. 1. To build a MRG signature for predicting the prognosis of BC patients, 164 mRNAs were obtained after screening overlapping MRGs and mRNAs the BRCA dataset from TCGA. Thereafter, we included the 164 MRGs in univariate Cox regression analysis and identified 15 genes significantly associated with OS (Fig. 2A). Univariate Cox analysis of genes with *p* values < 0.05 was performed by LASSO-Cox regression analysis to select hub genes, and 12 genes were selected for further multivariate Cox regression analysis (Figs. 2B and 2C). Ultimately, multivariate Cox regression analysis was employed to reduce the number of genes from 12 to 9, as follows: neurogenic locus notch homolog protein 1 (NOTCH1), protein tyrosine phosphatase 4A3 (PTP4A3), matrix metallopeptidase 13 (MMP13), F2R-like trypsin receptor 1 (F2RL1), metastasis-associated in colon cancer 1 (MACC1), EZR, neural precursor cell expressed, developmentally downregulated 9 (NEDD9), Phosphatidylinositol-4,5-Bisphosphate 3-Kinase Catalytic Subunit Alpha (PIK3CA), and C-C motif chemokine receptor 7 (CCR7) (Fig. 2D). As expected, a meaningful correlation between these nine MRGs and distant metastasis-free survival (DMFS) was validated using the KM plotter online database (Suppl. Fig. 1).

**Figure 3:**
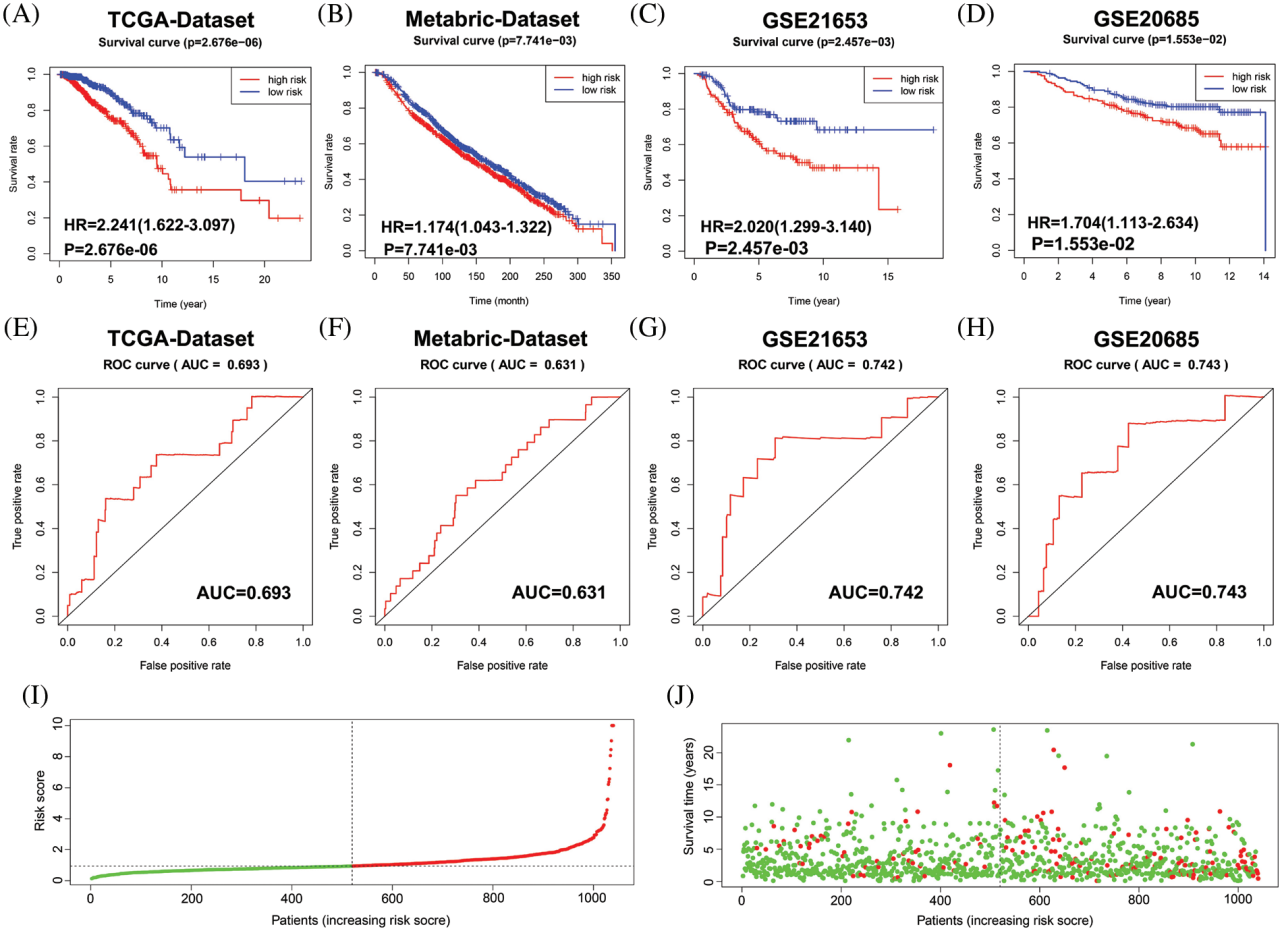
Evaluation of the performance of the 9 MRG signature in TCGA-BRCA cohort, METABRIC and two GEO datasets. (A–D) Kaplan–Meier survival curve analysis described the significant survival difference between the high-risk group and the low-risk group in TCGA, Metabric and two GEO datasets, respectively. (E–H) Time-dependent ROC curves of the 9 MRG signature for predicting the 5-year OS in the TCGA, METABRIC and two GEO datasets. (I) Distribution of risk scores in high-and low-risk groups of BC patients in TCGA dataset. (J) Distribution of survival status in BC patients with different risk scores, red dots mean people who were already dead, green dots mean people who were still alive.

Next, the final nine-gene signature formula was calculated for each patient in TCGA datasets, as follows: risk score = (NOTCH1 × 0.025856067 + PTP4A3 × 0.016839163 + MMP13 × 0.011252209 + F2RL1 × 0.061627176 + MACC1 × 0.054689802 + EZR × 0.003327923 + NEDD9 × −0.070163692 + PIK3CA × 0.019899521 + CCR7 × 0.014898432). Based on the median value of the risk score, patients in the cohort from TCGA were stratified into low- and high-risk groups, and allocations of the risk score and dot plot of survival status indicated poorer prognosis for BC patients with high risk (Figs. 3I and 3J). The Kaplan‒Meier survival curves revealed that TCGA dataset patients with high risk scores had high mortality and short survival rates (Fig. 3A). Similar trends were observed for OS in METABRIC (Fig. 3B) and two GEO (Figs. 3C and 3D) datasets. Then, we used a time-dependent ROC curve to evaluate the sensitivity and specificity of the risk scoring model for 5-year survival.

The AUC was 0.693 in TCGA (Fig. 3E), 0.631 in METABRIC (Fig. 3F), 0.742 in GSE21653 (Fig. 3G), and 0.743 in GSE20685 (Fig. 3H). These results suggest that the nine-MRG signature can effectively determine the prognosis of BC patients.

### Assessment of the correlation between the risk model and clinicopathological characteristics

**Figure 4:**
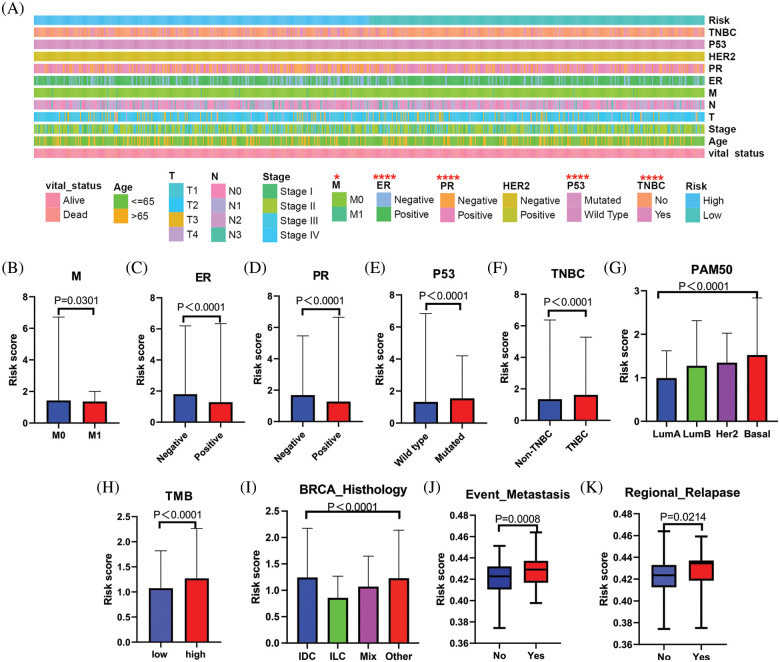
Relationship between the clinical features and MRG signature. (A) The strip chart shows the distributional differences of clinical features between high- and low-risk groups. (B) The riskscores were different based on different differences in the (B) M stage, (C) ER status, (D) PR status, (E) P53 status, (F) TNBC grade, (G) PAM50, (H)TMB, (I) BRCA pathology, (J) metastatic events, (K) regional relapse for BC patients.

To evaluate the association between the BC patient risk score and clinical characteristics, an overview strip chart of differences in clinicopathological characteristics between the low- and high-risk groups of all samples is shown in Fig. 4A. The results revealed significant differences in M stage (Fig. 4B), ER status (Fig. 4C), PR status (Fig. 4D), P53 status (Fig. 4E), and TNBC status (Fig. 4F). As shown in the box plot, the PAM50 tumor subtype (Fig. 4G), TMB (Fig. 4H), BRCA histology (Fig. 4I), metastatic events (Fig. 4J) and regional relapse (N stage) (Fig. 4K) also correlated significantly with the risk score.

### Stratification analyses

**Figure 5:**
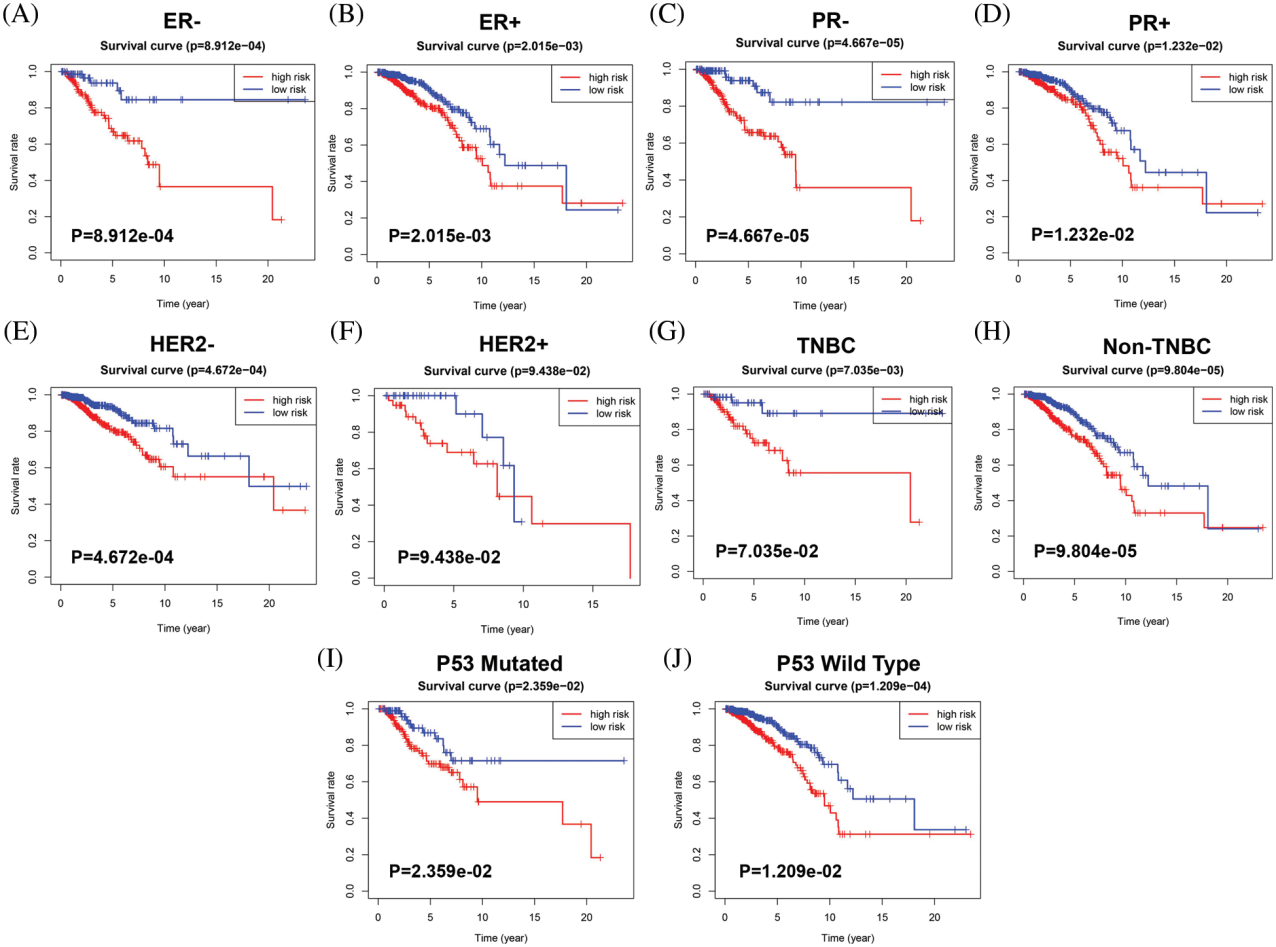
Stratified analysis of the prognostic significance of risk models in different clinical subgroups. Based on the risk score model, stratified survival analysis showed the OS rates in patients with different ER status group (A and B), PR statuses (C and D), and HER2 statuses (E and F), and molecular subtypes (G and H), and P53 statuses (I and J).

Furthermore, stratified analysis of clinicopathologic features revealed that BC patients in the high-risk group had a significantly shorter OS period in several strata, such as ER status (ER− or ER+) (Figs. 5A and 5B), PR status (PR− or PR+) (Figs. 5C and 5D), HER2 status (HER2− or HER2+) (Figs. 5E and 5F), TNBC (non-TNBC or TNBC) (Figs. 5G and 5H), and P53 status (P53 mutated type or P53 wild-type) (Fig. 5I and 5J). These results suggest that our 9 MRG signature is powerful for predicting the survival period of BC patients with different hormone receptor statuses, P53 statuses and TNBC grades.

### The metastasis-related gene signature is an independent factor

**Figure 6:**
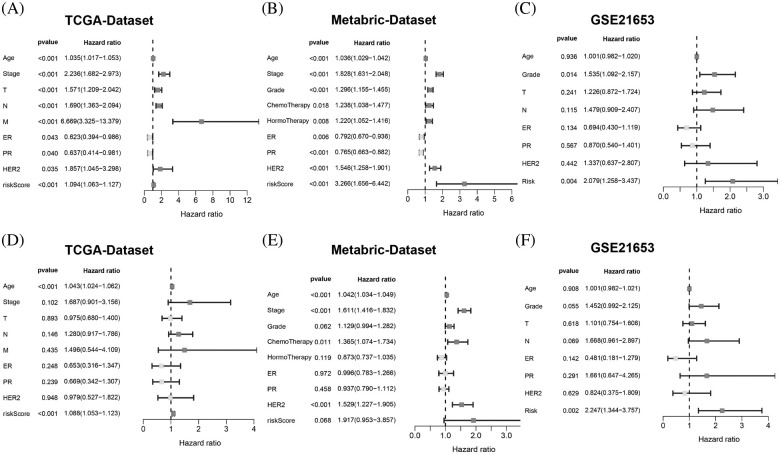
The MRG signature was able to serve as an independent prognostic factor in BC patients. (A and D) Univariate and multivariate Cox-regression analyses of the correlation between risk score, age, stage, T stage, N stage, M stage, ER status, PR status, HER2 status and OS in TCGA-BRCA cohort. (B and E) Univariate and multivariate Cox-regression analyses of the correlation between the risk score, age, grade, chemotherapy, hormotherapy, ER status, PR status, HER2 status and OS in METABRIC cohort. (C and F) Univariate and multivariate Cox-regression analyses of the correlation between the risk score, age, grade, ER status, PR status, HER2 status and OS in the GSE21653 cohort.

To verify whether the risk score acts as a prognostic index irrespective of other clinical features, we performed univariate and multivariate Cox regression analyses using multiple datasets. The results showed that the risk score alone was able to evaluate prognosis in univariate Cox regression analysis in the three datasets (Figs. 6A–6C). It was also an obvious predictive factor for prognosis after eliminating the influence of other characteristics in the datasets from TCGA (Fig. 6D) and GSE21653 (Fig. 6F) but not that from METABRIC (Fig. 6E).

### Construction and validation of a nomogram

**Figure 7:**
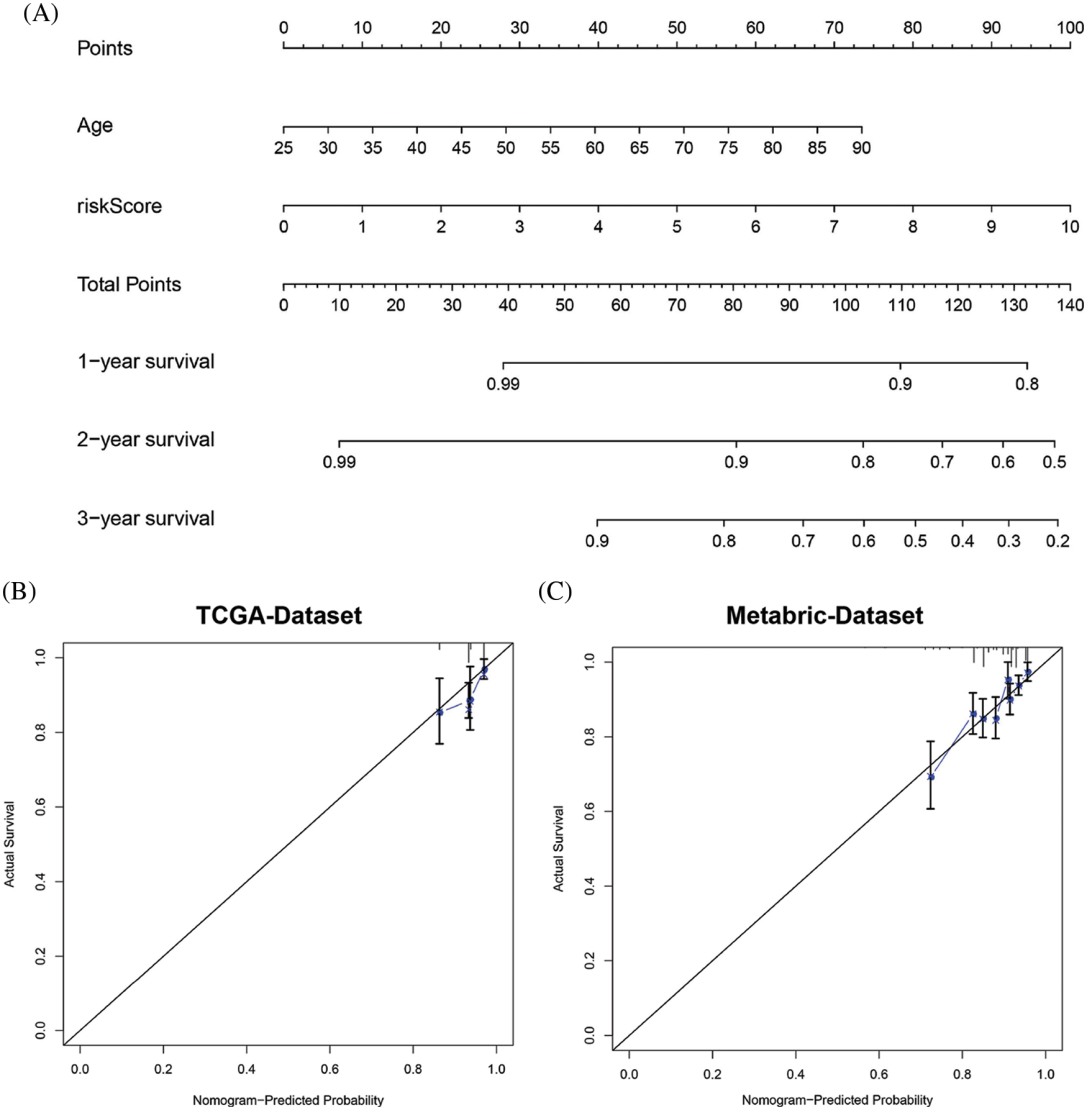
Establishment and evaluation of the nomogram model. (A) Prognostic nomogram incorporating the MRG signature predicting the 1-, 2- and 3-year overall survival of BRCA cohort. (B) Calibration plot of the nomogram for predicting the probability of OS at 1, 2, and 3 years in the TCGA-BRCA cohort. (C) The calibration plot of the nomogram for predicting the probability of OS at 1, 2, and 3 years in the Metabric cohort.

To determine the predictive efficacy of the MRG signature, we constructed an OS nomogram at 1-, 2- and 3-year by integrating the risk score with age in TCGA datasets. Results showed that shorter OS happened in older age and higher riskscores (Fig. 7A). Furthermore, the calibration curve for the predicted 3-year survival probability revealed that the predicted curve was very close to the ideal curve (Figs. 7B and 7C). This result suggests good predictive efficiency of the model.

### Functional analysis of the prognostic model

To further identify biological processes and Kyoto Encyclopedia of Genes and Genomes KEGG pathways associated with the risk signature, we performed GSEA for high- and low-risk patients classified by the risk score.

**Figure 8:**
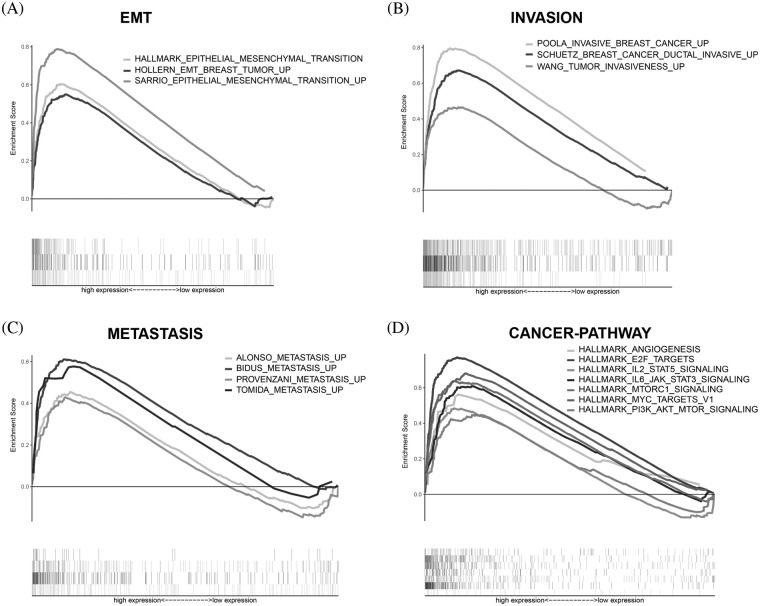
GSEA. (A) EMT-related gene sets were significantly enriched in the high-risk group. (B) Invasion-related gene sets were significantly enriched in the high-risk group. (C) Metastasis-related gene sets were significantly enriched in the high-risk group. (D) Cancer-related signaling pathways were significantly enriched in the high-risk group.

As expected, the high-risk score group showed obvious enrichment in EMT-related gene sets (Fig. 8A), invasion-related gene sets (Fig. 8B) and MRG sets (Fig. 8C). Interestingly, the high-risk score group also comprised significant enrichment of unfavorable cancer-related hallmark gene sets (Fig. 8D), such as “ANGIOGENESIS”, “E2F_Target”, “IL2_STAT5 signaling”, “IL6_JAK_STAT3 signaling”, “MTORC1 signaling”, “MYC_TARGETS_V1” and “PI3K_AKT_MTOR signaling”.

### Relationship between immune features, the stemness index and risk signature

**Figure 9:**
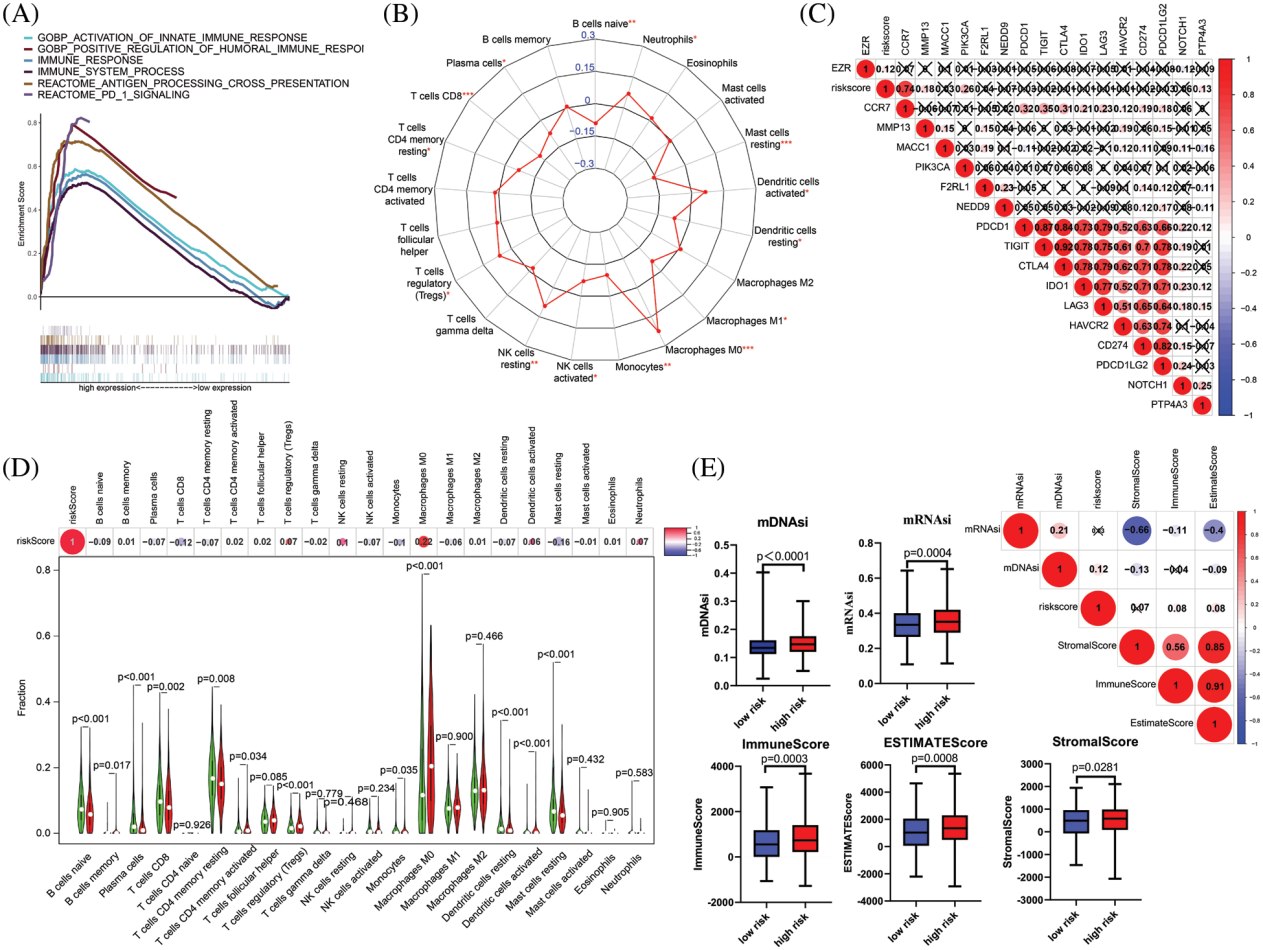
Tumor immunity and cancer stemness analysis of the 9 metastasis-related genes signature in TCGA-BRCA cohort. (A) Immune-related gene sets significantly enriched in the high-risk group identified by GSEA. (B) Radar chart showing the correlation between the risk score and 22 immune cell types. (C) A heatmap showing the relationship between the risk score and the expression of immune checkpoint genes. (D) Violin plot of differences in various immune cell abundances between the high-and low-risk groups. (E) Heatmap and box plot showing the correlation between the risk score, TME and stemness index. *p < 0.05, **p < 0.01, ****p < 0.005.

The immune system has been shown to play key roles in the occurrence and development of tumors. We first performed GSEA to evaluate the relationship between the risk score and immune features. As depicted in Fig. 9A, some pivotal immune-related gene sets were enriched in the high-risk group, including “activation of innate immune response”, “positive regulation of humoral immune response”, “immune response”, “immune system process”, “antigen processing cross presentation”, and “PD-1 signaling”. Subsequently, we used CIBERSORT to further support the correlation between 22 immune cell types and the risk signature, with a remarkable correlation with the risk score for 14 immune cell types. Among them, neutrophils, activated dendritic cells, M0 macrophages, gamma delta NK cells, and regulatory T cells were correlated positively with the risk score. In contrast, naive B cells, resting mast cells, resting dendritic cells, M1 macrophages, monocytes, and activated NK cells were negatively correlated with the risk score (Fig. 9B). The Wilcoxon-rank sum test was then performed to explore the significant differential distribution of 22 immune cell types in the low-risk group and high-risk group, and the results are presented as a violin plot in Fig. 9D. Although the 12 immune cell types were significantly different between the two groups, the risk signature showed no significant correlation with the eight immune checkpoint molecules (Fig. 9C). As previous studies have confirmed that cell stemness is a prerequisite for cancer invasion and metastasis, we further examined the correlation between the stemness index and the risk signature. As expected, both mDNAsi and mRNAsi were observed to be significantly higher in the high-risk group than in the low-risk group. Based on the ESTIMATE algorithm, the high-risk group had significantly higher ESTIMATE scores, immune scores and stromal scores than the low-risk group. A correlation heatmap analysis showed that the risk score correlated significantly positively with the mDNAsi, Immunescore and ESTIMATE scores (Fig. 9E).

### Rac1 and RhoA signaling mediates EZR-induced cell migration

**Figure 10:**
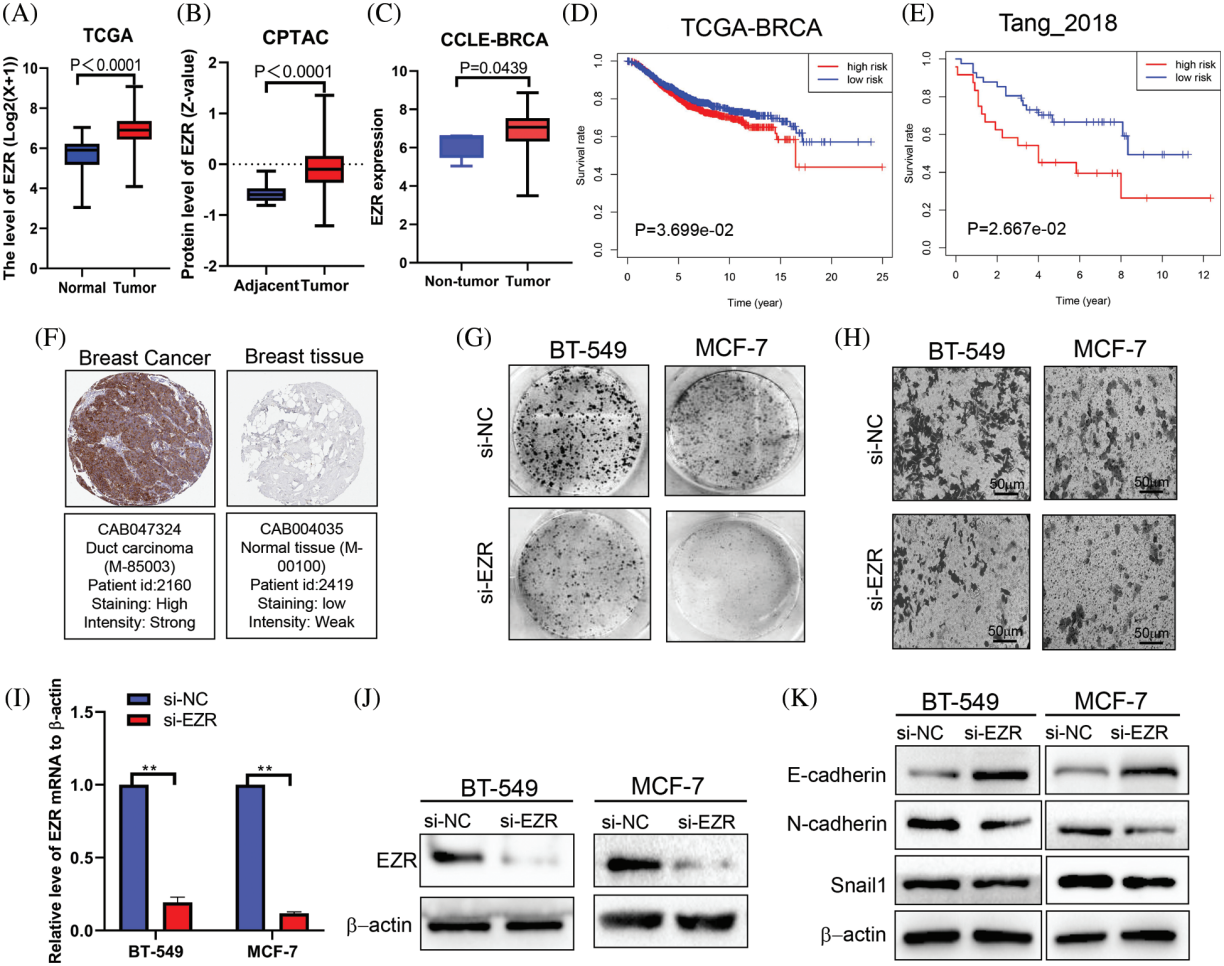
The effect of EZR on cell proliferation, cell migration and EMT. (A) The RNA expression of EZR in BC tissues (N = 1102) and normal tissues (N = 113) based on TCGA-BRCA dataset, *p* < 0.0001. (B) EZR protein expression in BC tissues (N = 133) and normal tissues (n = 18) in the CPTAC data, *p* < 0.0001. (C) The expression of EZR in five normal breast cell line and 63 BC cell lines was assessed by CCLE-dataset, *p* = 0.0439. (D) Kaplan-Meier survival curve for the OS of patients in high EZR expression group and low EZR expression group according to the Kaplan–Meier plotter database (https://kmplot.com/analysis/); *p* = 0.0369. (E) Kaplan-Meier survival curve for the OS of patients in high EZR expression group and low EZR expression group according to a proteomic data, *p* = 0.0266. (F) Immunohistochemistry of the EZR in BC tissues and normal tissues based on the HPAD. (G) Representative images of the colony formation assay were shown in BT-549 and MCF-7 cells after transfection with si-NC or si-EZR. (H) Cell migration was assessed with a Transwell assay after the knockdown of EZR in two BC cell lines. (I and J) The suppressive efficacy of si-EZR was confirmed by using qRT-PCR and western blotting assays. (K) Assessment of the effect of EZR on EMT in two BC cell lines by using western blotting assays. **p < 0.01.

**Supplemental Figure 2:**
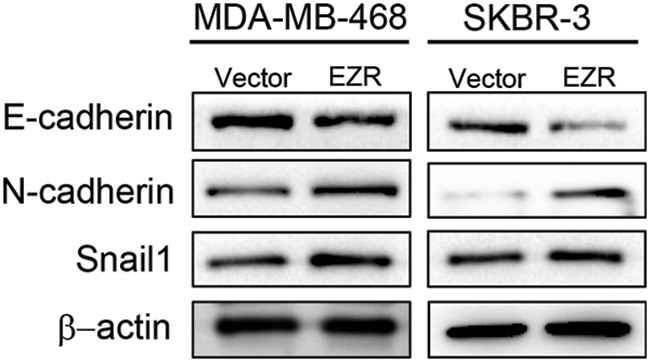
The effect of EZR overexpression on the EMT process in two breast cancer cell lines.

**Supplemental Figure 3:**
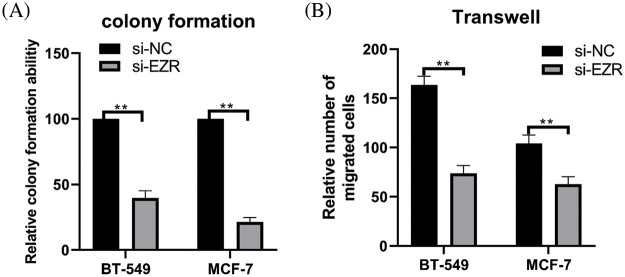
The effect of EZR knockdown on the colony formation assay and Transwell assay.

Among the nine MRGs, we selected EZR for further analysis of biological function and regulatory mechanisms. In TCGA, the expression of EZR was significantly upregulated in malignant tissues compared with adjacent normal tissues (Fig. 10A). Consistent with this finding, upregulated EZR was found in CPTAC datasets (Fig. 10B) and at the cellular level in CCLE datasets (Fig. 10C). We also examined the prognostic significance of EZR in BC patients through the Kaplan‒Meier plotter online database. Kaplan‒Meier survival curve analysis showed that high EZR expression at both RNA (Fig. 10D) and protein (Fig. 10E) levels predicted shorter OS, and the HPAD IHC data confirmed higher expression of EZR at the protein level in BC (Fig. 10F). To further analyze the biological functions of EZR in BC cells, we first knocked down EZR expression in two BC cell lines (Figs. 10I and 10J). In subsequent cell function experiments, we found that EZR knockdown significantly inhibited clonogenic ability (Fig. 10G), cell migration (Fig. 10H) and EMT (Fig. 10K). The enforced expression of EZR generated the opposite results in the EMT assay (Suppl. Fig. 2). Quantitative data of colony assay and transwell assays were shown in Suppl. Fig. 3.

**Figure 11:**
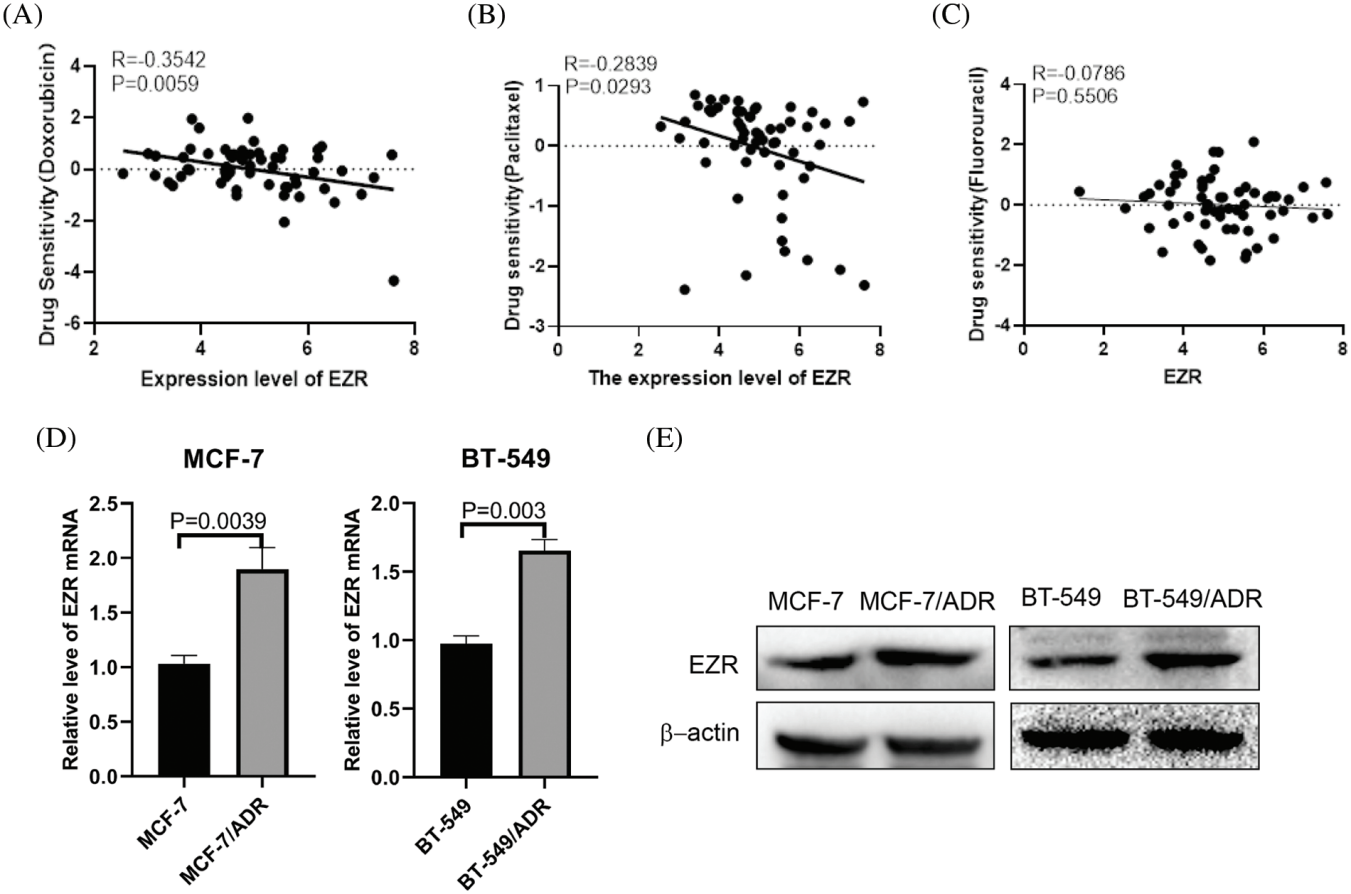
Effect of EZR on Doxorubicin chemoresistance in BC cells. (A) correlation between tumor cell line sensitivity to Doxorubicin and mRNA expression levels of EZR. R = −0.3542, *p* = 0.0059. (B) Correlation between tumor cell line sensitivity to Paclitaxel and mRNA expression levels of EZR. R = −0.2839, *p* = 0.0293. (C) Correlation between tumor cell line sensitivity to Fluorouracil and mRNA expression levels of EZR. R = −0.0786, *p* = 0.5506. (D) Q-PCR showing different levels of EZR mRNA expression in MCF-7 or BT-549 and MCF-7/ADR cells or BT-549/ADR cells. (E) Western blotting showing that different levels of EZR protein expression in MCF-7 or BT-549 and MCF-7/ADR cells or BT-549/ADR cells.

In addition, we used CellMiner, a web-based suite of bioinformatics tools designed to explore the drug sensitivity in the NCI-60 cell lines to mine the significantly associated drugs related to the transcription level of EZR [25]. Interestingly, the expression level of EZR was significantly negatively correlated with the drug activity of Doxorubicin (Fig. 11A) and Paclitaxel (Fig. 11B) but not Fluorouracil (Fig. 11C). To further investigate the effect of EZR on the cytotoxicity of doxorubicin in breast cancer cell lines. The expression level of EZR mRNA and protein were measured by using Western blotting and q-PCR. MCF-7/ADR cells and BT-549/ADR cells expressed higher level of EZR than wild-type two BCcells (Figs. 11D and 11E). Based on the above data, we speculated that inhibition of EZR expression may increase the sensitivity of BC cells to doxorubicin.

**Figure 12:**
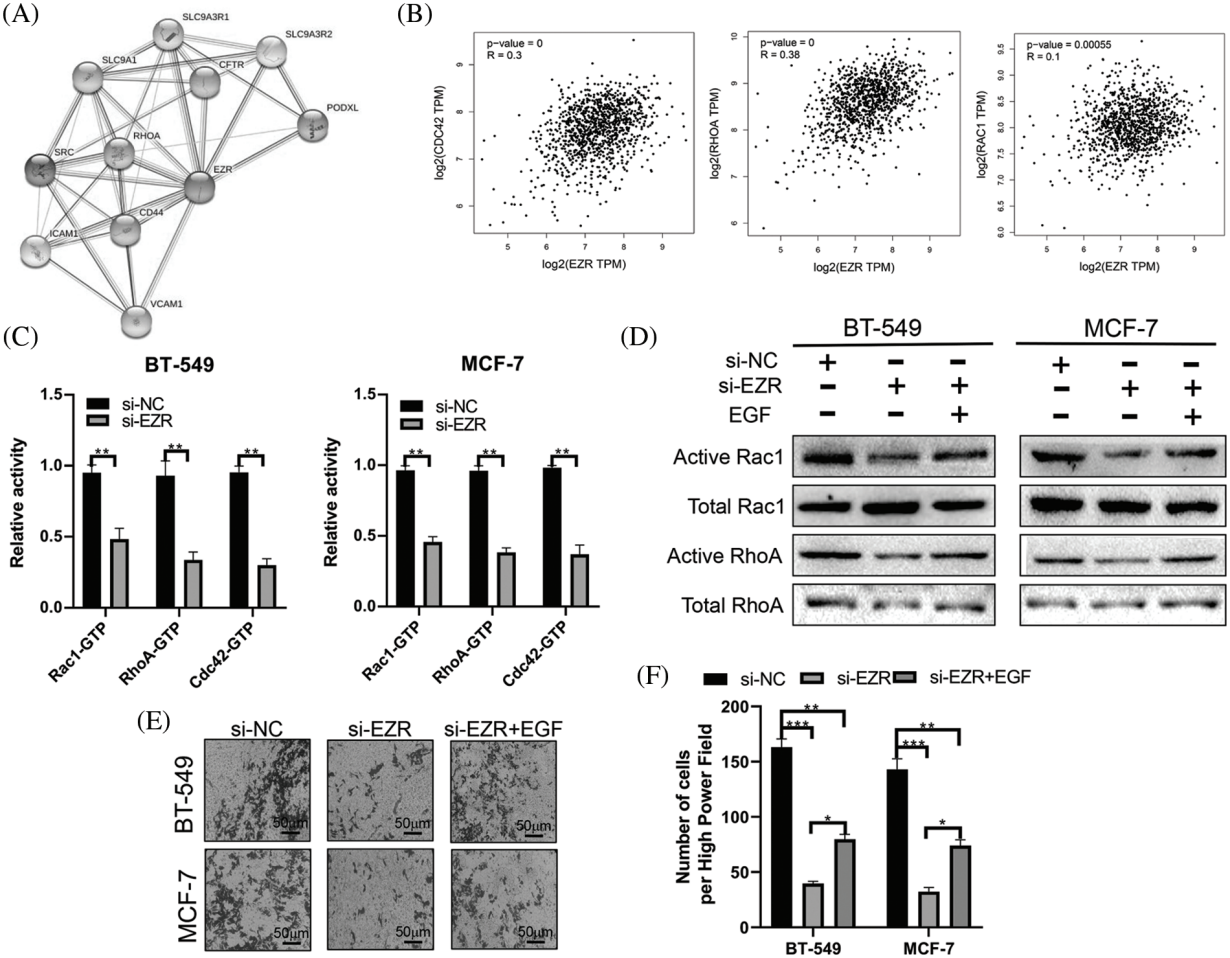
The effect of EZR on the RAC1/RhoA signaling pathway. (A) PPI network of EZR detected *in silico* using the STRING database. (B) EZR was predicted to have a positive correlation with cdc42, RhoA and Rac1 in BC using GEPIA. (C) The effect of EZR knockdown on the Rho GTPase guanine nucleotide binding status of Rac1, RhoA and Cdc42 was examined in two BC cells line. (D) Knockdown of EZR decreased Rac1/RhoA levels in BT-549 and MCF-7 cells, as detected by western blotting. (E–F) A Transwell assay showing the migration power of BT-549 and MCF-7 cells using EZR knockdown, or si-EZR combined with EGF. *p < 0.05, **p < 0.01, ***p < 0.005.

Finally, we used STRING (http://string-db.org/cgi/input.pl) to construct a PPI network for EZR. A total of 10 proteins were predicted to directly interact with EZR (Fig. 12A), and RhoA was found to correlate positively with EZR in the BRCA dataset from TCGA. In addition, the other two most studied members of the Rho GTPase family, namely, Rac1 and CDC42, were significantly positively associated with EZR in BC (Fig. 12B). Taking previous studies into consideration, the Rho family of GTPases plays an important role in the regulation of F-actin assembly and cell migration. To verify whether the biological function of EZR is exerted through the Rac1/RhoA/cdc42 pathway, the effect of EZR expression on this activity was assessed in EZR-knockdown BT-549 and MCF-7-cell lines using GTPase activity assays (Fig. 12C). A similar effect was illustrated in GST-TRBD and GST-PBD pull-down assays, in which RhoA and Rac1 activities were decreased basally following si-EZR treatment in two BC cell lines. EGF stimulation partially reversed the inhibitory effect of EZR knockdown on the activities of RhoA and Rac1 (Fig. 12D). These results show that si-EZR inhibited the activities of RhoA, Rac1 and Cdc42. Finally, to implicate these downstream pathways in the regulation of the migration of BC cells, we treated two BC cell lines with si-EZR and EGF and assessed effects on migration using a Transwell migration assay. We found that reducing EZR expression significantly inhibited cell migration and that EGF treatment partially reversed this inhibition (Figs. 12E and 12F). Taken together, these findings show that EZR may play a oncogenic role in BC through the Rac1/RhoA/cdc42 pathway.

## Discussion

Metastasis is responsible for 90% of cancer-associated mortalities in BC and thus has become the most lethal behavior of BC. In general, patients with localized BC may experience 5-year survival as high as 90%, whereas the same rate for metastatic BC may be as low as 25%. In addition, metastasis is arguably the most robust cause of treatment failure in BC. At the cytomolecular level, metastatic BC differs significantly from its in situ origin. Therefore, exploring the molecular mechanisms underlying BC metastasis is beneficial for identifying candidate diagnostic and therapeutic targets for metastasis.

With the development of high-throughput technologies, some candidate targets have been identified and indirectly or directly linked to metastasis in BC. Some MRG signatures have also been reported. For instance, Xie et al. identified a four-mRNA metastasis-related prognostic signature using four GEO datasets that was useful for predicting the disease-free survival DFS of BC patients [11]. Sui et al. reported a multidimensional prognostic signature including four genes and one lncRNA that was able to accurately subdivide lymph node metastasis status in BC [26]. Similar results have also been observed in other cancers, such as gastric cancer, hepatocellular carcinomas, and colorectal cancer.

In this study, we identified a 9-MRG signature. Among these genes, PTP4A3, MMP13, F2RL1, MACC1, EZR and CCR7 were increased in BC tissues compared with normal breast tissues, whereas NEDD9, NOTCH1 and PIK3CA were decreased, suggesting differential roles of the 9 MRGs in the metastatic process. Survival analysis showed that high NEDD9 expression was associated with longer DMFS in patients with BC. In contrast, high expression of the other 8 MRGs predicted shorter DMFS. Compared to the other seven genes, MMP13 and NOTCH1 have been the most studied in BC, especially regarding invasion and metastasis. Notch1 is a well-known oncogene driver of metastasis in a variety of tumors. Many early studies found that NOCTH1 enhances the metastatic ability of BC cells and is positively associated with axillary lymph node metastasis in BC patients [27]. MMP13 plays a critical role in the metastasis of tumor cells through the degradation of extracellular matrix proteins. Additionally, MMP13 is significantly upregulated in metastatic and recurrent BC tissues and promotes lung metastasis in BC [28]. The protein encoded by the PTP4A3 gene belongs to the protein-tyrosine phosphatase family, which mainly stimulates the transformation of cells from G1 to S phase during mitosis. The role of PTP4A3 associated with cell invasion and cancer metastasis has been extensively studied in other cancers but has seldom been reported in BC [29]. Only one study showed that phosphatase PTP4A3 promotes cell growth and G1-S cell cycle progression in TNBC cells [30]. F2RL1 (also named PAR2) is a member of the unique G-protein-coupled receptor subfamily and is expressed abundantly in various malignancies. Some previous studies have confirmed the robust association of PAR2 with metastasis in BC [31]. MACC1 is a key regulator of the hepatocyte growth factor (HGF) receptor and has mainly been identified as an independent prognostic factor for metastasis formation and metastasis-free survival in colon cancer. To date, studies on MACC1 in BC have mainly focused on analyzing its relationship with the clinicopathology and prognosis of patients, but there are few studies on its molecular mechanism in BC metastasis [32]. CCR7 is a G-coupled cheekiness receptor that was identified as a mediator of EBV effects on B lymphocytes. Thus, CCR7 is mainly involved in the migration/trafficking of immune cells. In our study, we also found that CCR7 correlated positively with multiple inhibitory immune checkpoint molecules. Regarding metastasis, recent studies have found that high expression of CCR7 correlates with lymph node metastasis and promotes cell invasion and migration processes through the AKT signaling pathway in BC [33]. The protein encoded by NEDD9 belongs to the CRK-associated substrate family and is a focal adhesion protein that is mainly involved in regulating cell attachment, migration and invasion. A recent study showed that NEDD9 exhibits prometastatic behavior in several solid tumors, including BC [34]. However, NEDD9 was found to be more highly expressed in normal breast tissues, and its high expression was associated with better DMFS, which contradicts its prometastatic behavior. PIK3CA is the most frequently mutated oncogene in BC, and mutations in this gene are known to activate the PI3K pathway [35]. Ezrin (encoded by EZR) is a cytoplasmic peripheral membrane protein that acts as a substrate of protein-tyrosine kinases. It also plays a role in cell adhesion, cell migration and organization and has been implicated in various human cancers [36,37]. One study showed that EZR expression was significantly upregulated in BC tissues and that its high expression predicts poorer prognosis [38]. Nevertheless, few studies have been conducted to investigate the molecular mechanism of EZR in BC metastasis. Here, we first analyzed differences in EZR expression in cancerous and normal breast tissues and the relationship between EZR and BC patient prognosis. The results are consistent with previous studies. Next, we explored the effect of EZR on cell function. Cell proliferation, migration and EMT assays confirmed that EZR plays an oncogenic role in BC cell lines. Our examination of molecular mechanisms revealed that EZR may be involved in BC cell proliferation and cell motility through the RhoA/RAC1 signaling pathway. Overall, current knowledge suggests that the roles of these nine prognostic MRGs in BC are worthy of further investigation.

Compared with early studies that established risk models to predict BC prognosis, we constructed a 9-MRG prognostic signature through a metastasis-associated gene set, which enabled us to gain more insight into the role of MRGs in BC tumorigenesis. Moreover, integrated analysis of the MRGs helped to dissect the molecular mechanisms involved in BC metastasis. Kaplan–Meier survival analysis showed significant prognostic differences between the high- and low-risk groups, and the survival nomogram confirmed the accuracy and sensitivity of the risk model in predicting the prognosis of BC patients. In addition, when combined with the commonly used TNM staging system, the MRG prognostic signature showed even better predictive ability in stratified analysis. Finally, we confirmed the broad application of the MRG prognostic signature for BC tumor characteristics by comprehensive analysis of risk scores and clinical features, tumor immunity and cancer stemness.

Compared with previous studies [11,39], we constructed a nine-MRG prognostic signature using TCGA-BRCA datasets and validated its accuracy in TCGA, METABRIC, and GEO datasets. This application of multiple platform datasets ensures the applicability of the risk model. In contrast to the purely bioinformatic analyses of previous studies, we deeply analyzed the biological functions and molecular mechanisms of EZR in BC cells. Nevertheless, there are still several limitations in the present study. First, as the 9-MRG prognostic signature was constructed and identified based on several relatively small cohorts, and a larger cohort is needed to verify the findings. Second, the samples used in this study were mostly from European and American populations, but the incidence and mortality rates of BC vary among ethnic groups, which can lead to inherent bias. Third, although we explored the biological function and specific mechanism of EZR in BC cells, its function needs in-depth exploration in animal studies and clinical practice. Therefore, future studies should take these factors into account to validate the current findings.

Taken together, we identified a 9-MRG signature that can serve as a prognostic indicator for BC. The MRG signature showed comparable performance in the prediction of patient prognosis and assessment of tumor immune cell infiltration, TME and cancer stemness. This risk model may facilitate the discovery of molecular biomarkers and therapeutic targets for BC patients, and the model has the potential to be widely used in clinical practice in the near future.

### Author contributions

Huijie Fan designed the study. Guodong Xiao and Feng Cheng performed the analysis. Guodong Xiao, Guodong Xiao, and Jing Yuan wrote the manuscript. Guodong Xiao performed cell experiments. Jing Yuan, Weiping Lu and Peili Wang contributed to preparing the figures and tables. JY and HJF revised the manuscript. All authors reviewed the manuscript and approved the final version.

### Availability of data and materials

Most of the datasets used and/or analyzed during the current study are publicly available data from TCGA, METABRIC, and Gene Expression Omnibus (GEO databases) (GSE21653 and GSE20685), The data used to support the findings of this study are available from the corresponding author upon reasonable request.

### Ethics approval

No additional ethical approval or informed consent was required in our study since all the raw data came from public data.
